# A large-scale reconfigurable multiplexed quantum photonic network

**DOI:** 10.1038/s41566-025-01806-x

**Published:** 2025-11-26

**Authors:** Natalia Herrera Valencia, Annameng Ma, Suraj Goel, Saroch Leedumrongwatthanakun, Francesco Graffitti, Alessandro Fedrizzi, Will McCutcheon, Mehul Malik

**Affiliations:** 1 Institute of Photonics and Quantum Sciences, Heriot-Watt University, Edinburgh, UK; 2 Present Address: Division of Physical Science, Faculty of Science, Prince of Songkla University, Songkhla, Thailand; 3 Present Address: Cyberhawk, Edinburgh, UK

**Keywords:** Quantum information, Quantum optics

## Abstract

The distribution of entanglement in quantum networks will enable the next generation of technologies in quantum-secured communications, distributed quantum computing and sensing. Future quantum networks will require dense connectivity, allowing multiple users to share entanglement in a reconfigurable and multiplexed manner, while long-distance connections are established through the teleportation of entanglement, or entanglement swapping. Although several recent works have demonstrated fully connected, local multi-user networks based on multiplexing, extending such networks to a global network architecture of interconnected local networks remains an outstanding challenge. Here we demonstrate the next step in the evolution of multiplexed quantum networks—a prototype global reconfigurable network in which entanglement is routed and teleported in a flexible and multiplexed manner between two local four-user networks. At the heart of our network is a programmable 8 × 8-dimensional multi-port circuit that harnesses the natural mode-mixing process inside of a multi-mode fibre to implement on-demand high-dimensional operations on two independent photons carrying eight transverse-spatial modes. Our circuit design allows us to break away from the limited planar geometry and bypass the control and fabrication challenges of conventional integrated photonic platforms. Our demonstration highlights the potential of this architecture for enabling large-scale, global quantum networks that offer versatile connectivity while being fully compatible with an existing communications infrastructure.

## Abstract (web summary)

A reconfigurable eight-user photonic network is realized by connecting two local four-user networks through a programmable 8 × 8-dimensional multi-port device. Multiplexed routing and swapping of qubit entanglement are demonstrated for all network configurations and channels.

## Main

Quantum networks enable the distribution and processing of quantum information between distant interconnected quantum nodes, with applications ranging from quantum communication to distributed quantum computing^1^. The distribution of entanglement over multi-user quantum network architectures in an efficient and scalable manner is of high importance. In addition to enabling next-generation quantum communication technologies such as device-independent quantum key distribution^2^, entanglement-based networks enable a distributed configuration that can bolster the performance of quantum computation^3,4^ and quantum metrology^5,6^.

**Fig. 1:**
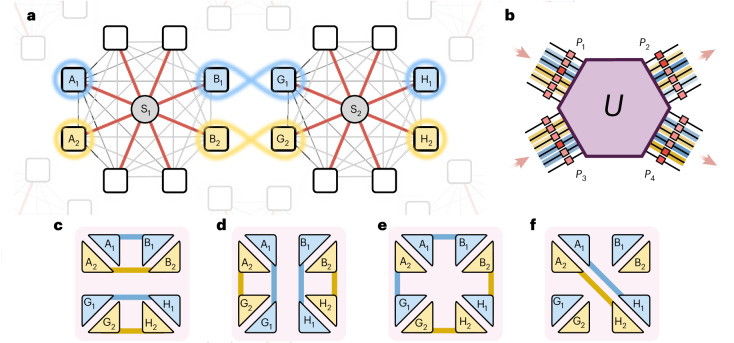
A global, multiplexed quantum network architecture. **a**, Two local, multiplexed quantum networks consisting of entanglement sources (S_1_ and S_2_) and sets of four users ({A_1_, A_2_, B_1_, B_2_} and {G_1_, G_2_, H_1_, H_2_}) are connected to form a global, eight-user multiplexed quantum network that can route and swap entanglement in a reconfigurable manner. **b**, Our programmable multi-port design consists of a complex medium (*U*) placed between four programmable phase planes (*P*_1_–*P*_4_) implementing an 8 × 8-dimensional multi-mode circuit that operates on two independent photons to implement entanglement routing and swapping operations. **c**–**f**, The global network can be reconfigured on demand to realize different network structures connecting pairs of users with qubit entanglement in several different configurations, including routing of entanglement for local network connectivity (**c**), global connectivity (**d**), local–global connectivity (**e**) and large-scale connectivity (**f**) through the multiplexed entanglement swapping between pairs of distant users A_1_H_1_ and A_2_H_2_.

Realizing a network in which entanglement can be shared between multiple users requires flexible and high-capacity platforms for generating and distributing entanglement. State-of-the-art network implementations have demonstrated the flexible routing of bipartite entanglement between multiple users via hybrid multiplexing in bulk and integrated platforms^7–12^. These demonstrations exploit an auxiliary photonic degree-of-freedom (such as wavelength) to multiplex several bipartite entanglement channels to realize fully connected, local quantum networks of up to eight users. The natural next step in the evolution of such networks is to interconnect multiple local, multi-user networks to realize a global multiplexed network in which distant users located at the edges of their respective local networks can share entanglement with each other (see Fig. 1a). A key obstacle to achieving such a global network is the lack of reconfigurable network devices that are capable of simultaneously routing and teleporting (or swapping) entanglement over multiplexed channels. Maintaining device performance while minimizing coupling losses becomes an important challenge as the number of photons and modes processed by such a device increases.

Conventionally, such network devices have been realized using the bottom-up approach, which relies on a planar mesh arrangement of a large number of carefully controlled two-mode interferometers^13,14^. Although integrated photonic circuit dimensionalities supporting up to 20 modes have been demonstrated recently^15^, increasing circuit complexity further will require overcoming many obstacles in circuit design and fabrication. Thermal management of the large number of components limits current performance and hinders further scalability^16^. A further hurdle is presented by the difficulty of efficiently coupling single photons from free-space or fibre links into an integrated circuit.

In this context, complex scattering media such as multi-mode fibres (MMFs) have recently emerged as promising candidates for both the transport and manipulation of quantum states of light^17,18^. Light scattering inside of an MMF can be described as a unitary and random linear optical process in a very high-dimensional space. Although this effect can be detrimental to the correlations of an entangled state, it can be reversed via knowledge of the fibre transmission matrix^19^, enabling the transport of high-dimensional spatial entanglement through a complex channel^20^. Using inverse-design techniques, the light-scattering process inside of a complex medium can also be exploited to achieve high-dimensional programmable optical circuits by placing the medium (*U*) between controllable phase planes (*P*) (Fig. 1b). This alternative top-down approach simplifies the circuit architecture by separating the control layer from the mixing layer by using a large number of auxiliary modes, achieving fully programmable high-dimensional quantum gates^21^. This approach also allows one to break out of the restrictive planar geometry of photonic integrated circuits and access the full transverse-spatial photonic degree-of-freedom distributed volumetrically. Finally, using waveguides for both transporting and manipulating quantum states of light bypasses the problem of coupling losses between optical links and photonic chips. In a recent work we demonstrated how an MMF-based circuit can be used to manipulate a single photon in its high-dimensional spatial structure, and function as a generalized, programmable multi-outcome measurement device for manipulating and certifying high-dimensional entanglement^21^.

Here we build on the top-down approach to implement a programmable 8 × 8-dimensional multi-port device and use it to develop a prototype global multiplexed quantum network architecture that routes and swaps entanglement in a reconfigurable and multiplexed manner between two local networks of four users each (Fig. 1a). Two local multi-user quantum networks are implemented via independent entanglement sources (S_1_ and S_2_) that distribute qubit entanglement to four sets of users in each network ({A_1_, A_2_, B_1_, B_2_} and {G_1_, G_2_, H_1_, H_2_}). A reconfigurable multi-port device (Fig. 1b) is implemented by placing an MMF (which is represented by the unitary operation *U*) between four programmable phase planes (*P*_1_–*P*_4_). The multi-port is used to connect the two local networks and realize a reconfigurable global network that can route and swap entanglement between all eight users in a multiplexed manner. The entanglement structures shown in Fig. 1c–f illustrate this versatile functionality, where a link between users (triangles) represents a shared qubit-entangled state. For example, multiplexed qubit entanglement can be shared between user pairs A_1_B_1_, A_2_B_2_, G_1_H_1_ and G_2_H_2_ (Fig. 1c). The network can also be reconfigured to share qubit entanglement between user pairs A_1_B_1_, A_2_G_1_, B_2_H_1_ and G_2_H_2_ (Fig. 1e). Importantly, the multi-port device can also implement multiplexed entangling operations on independent input photons, enabling entanglement to be swapped between distant user pairs A_1_H_1_ and A_2_H_2_ (Fig. 1f).

This demonstration showcases the potential of complex-media-based platforms for realizing large-scale and flexible quantum network architectures. Below, we describe the operation of our programmable multi-port device in detail and present results quantifying the performance of our reconfigurable global quantum network.

## Programmable multi-port design

**Fig. 2:**
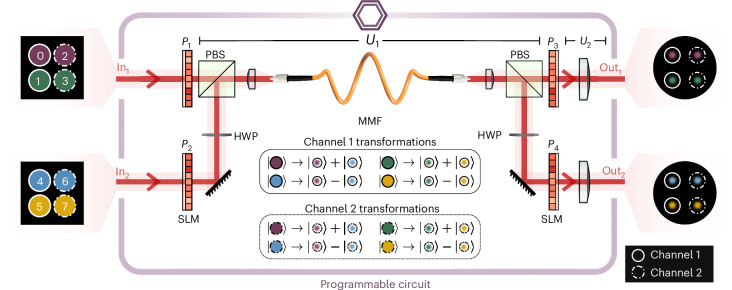
Programmable multi-port circuit design. A reconfigurable 8 × 8-dimensional multi-port circuit is implemented in a top-down manner^21^ by placing an MMF (represented by *U*_1_) between four programmable phase planes (*P*_1_–*P*_4_) displayed on SLMs. The circuit simultaneously operates on two input–output channels (1,2) each carrying four-dimensional spatial modes (labelled 0 to 7). The circuit implements transformations in the form ${\mathbb{T}}={U}_{2}({P}_{3}\oplus {P}_{4}){U}_{1}({P}_{1}\oplus {P}_{2})$ between input macro-pixel modes (left side) and focused Gaussian spots across the facet of the MMF (right side). The phase plane solutions (*P*_1_–*P*_4_) for a desired circuit transformation are obtained by using an inverse-design wavefront-matching algorithm^23,24^. The circuits programmed in this manner enable us to perform on-demand routing and interference operations on the eight input–output spatial modes to realize the entanglement structures shown in Fig. 1c–f. Here we show the transformation corresponding to a simultaneous Bell-state measurement across the two channels, which implements the multiplexed entanglement-swapping protocol shown in Fig. 1f. PBS, polarizing beam-splitter; HWP, half-wave plate.

We harness the complexity of the inter-modal coupling inside of a random scattering medium to construct a top-down programmable device that can implement arbitrary high-dimensional quantum gates operating on two independent photons. A linear circuit can be represented by a transformation ${\mathbb{T}}$ that maps a set of input modes to a set of output modes. As depicted in Fig. 2, our design consists of a 30-cm-long MMF (Thorlabs GIF625) acting as a large, ambient mode-mixer, placed between four phase planes implemented on programmable spatial light modulators (SLMs). The circuit’s transformation can be decomposed to ${\mathbb{T}}={U}_{2}({P}_{3}\oplus {P}_{4}){U}_{1}({P}_{1}\oplus {P}_{2})$, where *P*_1_–*P*_4_ represent the reconfigurable phase planes; *U*_1_ describes the transmission through the MMF and associated coupling optics; and *U*_2_ corresponds to the optical system between phase planes *P*_3_ and *P*_4_, and the detection system.

The circuit is designed by first characterizing the MMF transmission matrix *U*_1_ across all input and output modes. This is performed with classical illumination and a physics-based neural network approach that describes the optical system of the experiment^22^. With complete knowledge of the scattering process in the MMF, desired target circuits ${\mathbb{T}}$ can be inverse-designed using iterative wavefront-matching optimizations of input and output optical fields propagating forwards and backwards through phase planes *P*_1_–*P*_4_ (refs. ^23,24^). The phase plane solutions obtained in this manner are implemented via holograms displayed on the programmable SLMs.

Our multi-port circuit implements high-dimensional quantum operations in the photonic transverse-spatial degree-of-freedom. Here we are free to choose a suitable discretized spatial-mode basis, which may be dictated by the type of multi-mode waveguides used in the network. At the input of the multi-port, we choose the macro-pixel basis^25^, which comprises localized circular modes (Fig. 2, left). At the output of the multi-port (following a lens), we target modes randomly selected from a set of focused Gaussian spots distributed isometrically across the facet of the MMF (Fig. 2, right). These modes can be easily coupled to single-mode telecom fibres or to cores of a multi-core fibre^26^, allowing compatibility with existing or future telecom infrastructure. To utilize the full dimensionality of the MMF, we exploit the polarization-dependence in the mode-mixing process of the MMF and associate the two polarizations with the two input ports (In_1_, In_2_), into which we inject two independent photons. At the output of the multi-port, these two polarizations also define the two output ports (Out_1_, Out_2_) used for directing photons to different users.

At each input port, we construct a four-dimensional spatial-mode basis composed of macro-pixel modes ${\{\left\vert m\right\rangle \}}_{m}$, where *m* = {0, 1, 2, 3} labels the modes at input 1, and *m* = {4, 5, 6, 7} labels the modes at input 2. Each four-dimensional input set is split into two qubit subspaces, which are used as distinct channels for multiplexing qubit entanglement in the network. As such, the multi-port operates on four distinct qubit channels composed of eight input macro-pixel modes. At input 1, we define the two channels as ${\mathrm{Ch}}_{1}=\{\left\vert 0\right\rangle ,\left\vert 1\right\rangle \}$ and ${\mathrm{Ch}}_{2}=\{\left\vert 2\right\rangle ,\left\vert 3\right\rangle \}$; at input 2, we define them as ${\mathrm{Ch}}_{1}=\{\left\vert 4\right\rangle ,\left\vert 5\right\rangle \}$ and ${\mathrm{Ch}}_{2}=\{\left\vert 6\right\rangle ,\left\vert 7\right\rangle \}$. The same is done with the eight foci modes at the output ports to define a total of four output qubit channels.

The multi-port circuit enables coherent control over the composite eight-dimensional modal space of two input photons. When these photons are obtained from two independent local networks, the multi-port acts as an interconnect, enabling multiplexed entanglement routing and swapping operations across the two local networks. Reconfigurable connectivity is implemented by programming different 8 × 8 unitary operations using the SLMs, without the need for any changes to the optical set-up. For example, we can realize the entanglement structure shown in Fig. 1c with an identity ${\mathbb{T}}_{\rm{I}}$ gate. The entanglement structures shown in Fig. 1d,e are realized by programming the operations ${\mathbb{T}}_{\rm{X}}$ and ${\mathbb{T}}_{\rm{M}}$ (refer to Supplementary Information for the matrix representations of the circuits). As an example, here we define the operation ${\mathbb{T}}_{\rm{S}}$ that allows us to perform the multiplexed entanglement-swapping protocol between two pairs of distant users (Fig. 1f):

1 $${{\mathbb{T}}}_{\rm{S}}=\,\frac{1}{\sqrt{2}}\left[\begin{array}{cccccccc}1&0&0&0&1&0&0&0\\ 0&1&0&0&0&1&0&0\\ 0&0&1&0&0&0&1&0\\ 0&0&0&1&0&0&0&1\\ 1&0&0&0&-1&0&0&0\\ 0&1&0&0&0&-1&0&0\\ 0&0&1&0&0&0&-1&0\\ 0&0&0&1&0&0&0&-1\end{array}\right].$$

The operation of this 8 × 8 circuit is illustrated in Fig. 2. As can be seen, this circuit implements two simultaneous beam-splitter operations across the two input–output channels, mixing the eight input macro-pixel modes unitarily with each other. This serves as a multiplexed entangling gate, enabling parallel Bell-state measurements to be made on each channel (refer to Methods for further details on the multiplexed entanglement-swapping protocol).

## Global reconfigurable entanglement network

We demonstrate the operation of the programmable multi-port circuit by connecting two local, four-user multiplexed entanglement networks to realize a global multiplexed network of eight users (Fig. 1a) with several different network configurations (Fig. 1c–f). The local networks are implemented by generating high-dimensional spatial-mode entanglement in two nonlinear crystals (S_1_ and S_2_) via spontaneous parametric down-conversion (SPDC). Refer to Supplementary Information for further details on the preparation and characterization of the entanglement generated at each source.

Using the four-dimensional macro-pixel basis ${\{\left\vert m\right\rangle \}}_{m}$, each bipartite entangled state is multiplexed into two qubit entanglement channels and distributed to the four users in each local network. The users in local networks 1 and 2 are labelled as {A_1_, A_2_, B_1_, B_2_} and {G_1_, G_2_, H_1_, H_2_}. Each user is equipped with a detection system for performing projective measurements of arbitrary spatial modes using an SLM, single-mode fibre and single-photon detector (refer to Methods for a detailed description of the multi-port device and the entanglement measurement methods).

**Fig. 3:**
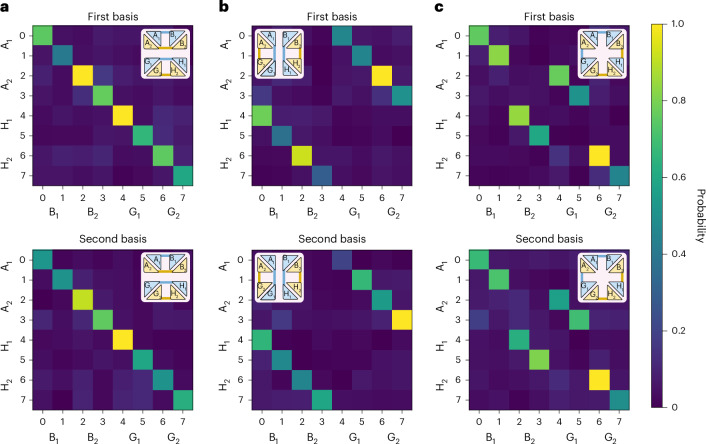
Multiplexed entanglement routing. **a**–**c**, We program 8 × 8-dimensional circuit operations that route qubit entanglement between eight users in three different global network configurations (insets). These include entanglement links between four local user pairs AB and GH (**a**), four global user pairs AG and BH (**b**), and four pairs consisting of both local and global user combinations (**c**). Normalized two-photon correlations measured in two MUBs are shown for each network configuration. By applying a suitable entanglement witness, we are able to certify the successful routing of entanglement in all three configurations. Source data

The programmable multi-port operates on two independent photons distributed from S_1_ to users B_1_, B_2_ and S_2_ to users G_1_, G_2_. We attain the three multiplexed entanglement structures shown in Fig. 1c–e by implementing the circuits ${\mathbb{T}}_{\rm{I}}$, ${\mathbb{T}}_{\rm{X}}$ and ${\mathbb{T}}_{\rm{M}}$*.* We quantify the quality of the network structures by measuring two-photon correlations between the eight users for each chosen multiplexed network configuration and applying suitable entanglement witnesses. The implementation of the first network structure via circuit operation ${\mathbb{T}}_{\rm{I}}$ is illustrated in Fig. 3a, where measurements in two mutually unbiased bases (MUBs) show correlations between local user pairs A_1_B_1_, A_2_B_2_, G_1_H_1_ and G_2_H_2_. The network is then reconfigured to attain the first global network structure via circuit operation ${\mathbb{T}}_{\rm{X}}$, with strong two-photon correlations observed between user pairs from different local networks: A_1_G_1_, A_2_G_2_, B_1_H_1_ and B_2_H_2_ (Fig. 3b). Finally, the global network structure corresponding to multiplexed entanglement links across both local and global networks is realized via the circuit operation ${\mathbb{T}}_{\rm{M}}$, with two-photon correlations observed between user pairs A_1_B_1_, A_2_G_1_, B_2_H_1_ and G_2_H_2_ (Fig. 3c).

Although Fig. 3 shows correlations in two MUBs, we also measure correlations in the third MUB of each two-dimensional subspace. This allows us to calculate the exact fidelity to a target entangled state via a fidelity witness^27^ and certify the quality of the multiplexed entanglement distribution in all network configurations. As shown in Table 1, we estimate the fidelities of the entangled states shared between all user pairs to the two-dimensional maximally entangled state. For circuit operation ${\mathbb{T}}_{\rm{I}}$, we obtain entangled state fidelities of 78.6% or higher, demonstrating the successful distribution of multiplexed qubit entanglement between all four local user pairs. The first global network configuration realized by circuit operation ${\mathbb{T}}_{\rm{X}}$ results in entangled state fidelities of 79.2% or higher, demonstrating that switching from a local to a global structure does not compromise the entanglement quality. Finally, the local–global configuration implemented by circuit operation ${\mathbb{T}}_{\rm{M}}$ also results in qubit entanglement being certified with a fidelity of 76.5% or higher, demonstrating the versatility of the multi-port circuit. We also test the ability of the multi-port circuit to route qubit and qutrit (*d* = 3) entanglement over a single channel (without multiplexing) in two different network configurations of four users. As shown in Supplementary Information, we able to certify successful routing of qubit and qutrit entanglement for both network configurations. The errors in the fidelities were calculated using a Monte Carlo simulation that takes into account Poissonian photon-counting statistics.

**Table 1:** The fidelities of the maximally entangled states shared in the multiplexed programmable network using eight-dimensional gates

	**Fidelities (%)**			
${{\mathbb{T}}}_{\rm{I}}$	A_1_B_1_	A_2_B_2_	G_1_H_1_	G_2_H_2_
	86.0 ± 1.0	78.6 ± 0.9	86.3 ± 0.9	79.2 ± 1.1
${{\mathbb{T}}}_{\rm{X}}$	A_1_G_1_	A_2_G_2_	B_1_H_1_	B_2_H_2_
	81.6 ± 1.0	79.2 ± 1.1	81.7 ± 1.2	80.8 ± 1.1
${{\mathbb{T}}}_{\rm{M}}$	A_1_B_1_	A_2_G_1_	B_2_H_1_	G_2_H_2_
	82.3 ± 1.0	81.2 ± 1.2	76.5 ± 1.2	81.4 ± 1.0
${{\mathbb{T}}}_{\rm{S}}$	A_1_H_1_		A_2_H_2_	
	77.1 ± 3.3		83.2 ± 2.7	

*Errors are reported to 1 s.d.

**Fig. 4:**
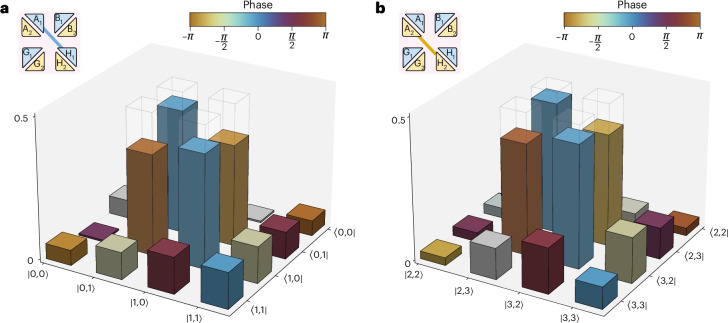
Multiplexed entanglement swapping. **a**,**b**, Reconstructed density matrices of the qubit-entangled states shared between distant user pairs A_1_H_1_ (**a**) and A_2_H_2_ (**b**), via the circuit operation implementing simultaneous Bell-state measurements on two multiplexed channels. The fidelities of the two-dimensional maximally entangled target states $\left\vert {\Psi }_{\rm{T}}\right\rangle$ (transparent bars) are 77.1 ± 3.3% for channel 1 and 83.2 ± 2.7% for channel 2, demonstrating the success of our entanglement-swapping protocol. The elements of the density matrices have their amplitude given by the height of the column bars, and their phase given by the colour. Source data

Finally, we implement the circuit operation ${\mathbb{T}}_{\rm{S}}$ to achieve multiplexed entanglement swapping between distant user pairs A_1_H_1_ and A_2_H_2_—connecting the edges of the two local networks with entanglement links. The swapping operation is conditioned on successful multiplexed Bell-state measurements between user pairs B_1_G_1_ and B_2_G_2_. We perform two sets of four-photon correlation measurements across the two network channels to certify the quality of this challenging network configuration. This allows us to characterize the swapped entangled states between the distant users via quantum state tomography (Methods). Figure 4 shows the reconstructed density matrices of the two entangled states shared between A_1_H_1_ and A_2_H_2_. The estimated states in each channel have fidelities to a maximally entangled target state of the form $\left\vert {\Psi }_{\rm{T}}\right\rangle =\frac{1}{\sqrt{2}}\left(\left\vert {0}_{\rm{A}}{1}_{\rm{H}}\right\rangle -{e}^{i\theta }\left\vert {1}_{\rm{A}}{0}_{\rm{H}}\right\rangle \right)$ of more than 77% (with the phase *θ* = 1.535*π* arising from the relative phase between input polarizations). This successfully demonstrates that our multi-port circuit is able to perform multiplexed entangling operations that are critical for interconnecting several independent local quantum networks.

We also test the stability of the programmable circuit over time (Supplementary Information) by repeatedly implementing all 8 × 8 bipartite routing operations and measuring the fidelity to the maximally entangled state of all distributed entangled states over a period of two weeks. During this time, the mechanical stability of the MMF is maintained with simple coupling stages or clamps to the optical table. We observe that the fidelity of the distributed entangled states remains well above the bound, without the need for realignment or recharacterization of the MMF transmission matrix (*U*), demonstrating the robustness of our circuit implementation.

## Discussion

We have demonstrated a reconfigurable multiplexed photonic network that connects two local four-user networks to realize a global eight-user network, allowing distant pairs of users to share entanglement in a multiplexed and flexible manner. Our network demonstration uses a unique multi-port device that exploits the large mode-mixing process inside of a MMF to implement high-dimensional operations on two independent photons carrying eight spatial modes. We achieve multiplexed routing and swapping of qubit entanglement, attaining fidelities exceeding 76% relative to the maximally entangled state for all network configurations and channels. We also demonstrate the routing of qutrit entanglement, highlighting the versatility our MMF-based device. To enable multiplexed operation, two-dimensional spatial-mode subspaces are used as distinct entanglement-distribution channels, allowing users to share multiple qubit-entangled states simultaneously. Moreover, our ability to perform programmable Bell-state measurements in parallel enables us to link four remote users by simultaneous entanglement swapping over two independent channels.

Our multi-port device enables efficient integration into existing communications infrastructure due to its straightforward coupling to optical fibre links. The stability of our system is maintained over weeks, and there is no need for special protective enclosures or recalibration of the mode-mixing process. With our top-down approach, the fidelity and size of the operations can be improved by a higher number of programmable phase layers within the circuit or a larger dimensionality in the auxiliary modal space offered by the MMF^21^. However, effects such as loss or modal dispersion become more prevalent in these regimes, and shorter fibre lengths or spectral-temporal control techniques may be necessary. Nevertheless, our device serves as a powerful approach towards realizing large-scale, multi-user entanglement networks with reconfigurable connectivity. As the number of modes and users increase, the characterization of such a device may require efficient process fidelity witnesses based on minimal measurements^28^. Future implementations of such a multi-port device could also explore entanglement in different high-dimensional spatial-mode bases^29,30^, as well as other photonic degrees-of-freedom such as time-energy^31^. Our implementation demonstrates the potential of complex-media-based platforms for realizing practical and noise-robust quantum network architectures^32^, as well as the control and distribution of large quantum-photonic states.

## Methods

### Experimental set-up

Two local, multiplexed four-user quantum networks are implemented through two independent pairs of photons that are highly dimensionally entangled in their transverse-spatial mode. The entangled photons are generated through a type-II SPDC at 1,550 nm in two identical non-linear periodically poled potassium titanyl phosphate crystals (1 mm × 2 mm × 5 mm).

A 30-cm-long graded-index MMF with a core diameter of 62.5 μm (Thorlabs GIF625) is used as a complex mode-mixer for implementing a top-down, inverse-design approach for programmable, multi-mode quantum circuits^21^. The fibre supports approximately 150 modes per polarization at 1,550 nm, and is placed between two programmable phase layers that comprise two phase planes each ({*P*_1_, *P*_2_} and {*P*_3_, *P*_4_}), which are implemented on two spatial light modulators (Hamamatsu X10468-08).

By enabling coherent control over the composite high-dimensional transverse-spatial modal space of the two input photons from independent local network states, the multi-port acts as an interconnect, enabling multiplexed entanglement routing and swapping operations across the eight-user global network.

Each of the eight users can perform single-outcome spatial-mode projections with the combination of programmable phase-screens implemented on SLMs, single-mode fibres (SMFs), and superconducting nanowire detectors^33^. Pairs of users {(A_1_,A_2_), (B_1_,B_2_), (G_1_,G_2_), (H_1_,H_2_)} use one phase-screen each to perform projective measurements in their respective two-dimensional subspace. For the classical characterization of the complex mode-mixing in the fibre and initial testing of the gates, a removable mirror is used to propagate laser light through the circuit, with an InGaAs camera (Allied Vision, Goldeye G-008 Cool TEC1) at the output.

Please refer to Supplementary Information for further details on—and a detailed figure of—the experimental set-up.

### Programmable optical circuit

Our top-down programmable optical circuit^21^ exploits random scattering inside of a MMF. The circuit is designed to operate on the macro-pixel basis on the input side, while the target output modes are randomly selected from the set of all possible focused spots (foci) distributed isometrically across the output facet of the MMF. Although our fibre supports approximately 150 spatial modes per polarization, we over-sample the fibre facet to improve the characterization of the transmission matrix.

The mode-mixing process inside the fibre is dependent on polarization. Light with horizontal polarization has a different transmission matrix than light with vertical polarization. Furthermore, input modes with a given polarization will exit the fibre in two different polarizations because the mode-mixing process does not preserve polarization. This polarization mixing allows us to take advantage of the full modal space offered by the MMF by defining the ports of the circuit according to polarization^18,34^. As shown in Fig. 2, we have two input (inputs 1 and 2) and two output (outputs 1 and 2) photon ports.

The screen of SLM_1_ is divided into two regions to be used as phase planes *P*_1_ and *P*_2_, allowing for the independent control of light entering from each input port. Although the signal photons (*s*_1_ and *s*_2_) are generated with the same polarization in their respective crystal, we rotate the polarization of one of the photons (not shown in the figure), superpose them with a PBS, and inject them into the MMF with orthogonal polarizations. Light coming out of the fibre is separated using a second PBS, with each output polarization reflecting on a different region of SLM_2_ to be used as phase planes *P*_3_ and *P*_4_, and directed to its corresponding detection stage.

### Detection and state analysis

Projective spatial measurements on idler photons (${\hat{\Pi }}_{\rm{a}}^{\mu }$) carrying spatial mode *a* in basis *μ* are implemented using SLM_3_, followed by coupling into SMFs that guide the photons to users A_1,2_ and H_1,2_. For the pair of signal photons passing through the circuit, phase planes *P*_1_–*P*_4_ are used to display the wavefront-matching solutions of the targeted operation, with phase planes *P*_3_ and *P*_4_ simultaneously performing projective spatial measurements on photons exiting the circuit. These photons are then coupled to SMFs that are guided towards users B_1,2_ and G_1,2_, and subsequently detected by superconducting nanowire single-photon detectors (Quantum Opus, Opus One, efficiency >85% at 1,550 nm). A counting logic (Swabian, TimeTagger Ultra) records coincidences between the eight users with a window of 200 ps.

We note that due to our users’ detection devices containing only one superconducting nanowire detector per node, we perform the tomography or witnesses for each state or channel separately, whereas equipping each user with their own detector would enable their multiplexed states to be recorded simultaneously or in parallel, with no reconfiguration of the programmable circuit.

When sending light from a classical source through the system, a removable mirror allows us to switch from single-photon detection to imaging of the output speckle patterns using an InGaAs camera without introducing changes to the circuit. A 400 mm lens focuses light reflecting from phase planes *P*_3,4_ onto the sensor of the camera, where images of the output facet of the fibre are taken for the characterization of the MMF transmission matrix *U*_1_ and the initial test of the circuit’s functionality.

### Characterization of the complex mode-mixer and the inverse design of the optical circuit

To characterize the transformation *U*_1_ across all input spatial and polarization modes, we swap the entangled photon sources at the input for a laser source (*λ* = 1,550 ± 3 nm) that is mode-matched to the photons entering the circuit. We then modulate the light input into the MMF via the two input ports corresponding to *s*_1_ and *s*_2_ by displaying random patterns *x*_1_ and *x*_2_ on input- and output-phase-planes *P*_1_/*P*_2_ and *P*_3_/*P*_4_, respectively, after which it is Fourier-transformed by a lens and measured by the camera. We use a multi-plane neural network^22^ to learn the transmission matrix of the MMF for each input polarization *U*_1_ by optimizing the following cost function

2 $$\mathop{\min }\limits_{{U}_{{1}_{i}}}\left\vert {y}_{i}-| {U}_{2}({x}_{{2}_{i}}\odot ({U}_{{1}_{i}}{x}_{{1}_{i}})){| }^{2}\right\vert ,$$

where *U*_2_ corresponds to the transformation by the lens. Stacking the transmission matrices for each polarization *U*_1_ allows us to reconstruct *U*_1_. This method enables us to bypass the need for an external reference with a machine-learning model that describes the optical system used in the experiment.

As the transmission matrices *U*_1_ are measured independently, we have no information on the relative phase between different input polarizations. Although this phase does not affect the performance of the circuit when routing or switching entanglement, it has an effect on the entanglement-swapping protocol where it introduces a phase on the state between users A_1,2_ and H_1,2_, which takes the form $\left\vert \Psi \right\rangle =\frac{1}{\sqrt{2}}\left(\left\vert 01\right\rangle +{e}^{i\varphi }\left\vert 10\right\rangle \right)$.

To ensure that the classical characterization of the spatial mode-mixing process inside of the fibre reproduces the behaviour of the SPDC photons, we use a system of telescopes for careful mode-matching between the laser source and the estimated spatial distribution of the multimodal SPDC emission^35^. To characterize the coupling loss in the circuit, we measure the coupling efficiency from the free-space single-mode laser source to the MMF to be above 76%.

With complete knowledge of the scattering processes in the mode-mixer, target circuits can be inverse-designed using phase pattern solutions for planes *P*_1_−*P*_4_ that are calculated with iterative wavefront-matching optimizations of input and output optical fields^21^. For this, input and output modes are generated from a chosen set of discrete spatial-mode bases and a target transformation ${\mathbb{T}}$. Each of the input modes is forward-propagated computationally, whereas each corresponding output mode is backward-propagated to each of the phase layers comprising of phase planes {*P*_1_, *P*_2_} and {*P*_3_, *P*_4_}. The phase of each of these planes is then updated to minimize the phase difference between the wavefront of the input and output modes. This process is repeated a few times until a convergence is observed. We then display the optimized phase-plane solutions *P*_1_−*P*_4_ in the physical set-up for it to function as an optical circuit.

### Entanglement swapping

An initial basic operation is to swap entanglement over one single channel per node. We consider two-dimensional input states of the form: $\left\vert \phi \right\rangle =\frac{1}{\sqrt{2}}\left(\left\vert 00\right\rangle +\left\vert 11\right\rangle \right)$, each produced by a different source. The complete initial four-photon state is then described as

3 $$\left\vert \Phi \right\rangle =\frac{1}{2}\left(\left\vert 0000\right\rangle +\left\vert 0101\right\rangle +\left\vert 1010\right\rangle +\left\vert 1111\right\rangle \right),$$

where each element of the ket corresponds to the state of the photons *s*_1_, *s*_2_, *i*_1_ and *i*_2_, respectively.

The entanglement-swapping operation acting on signal photons *s*_1_ and *s*_2_ is defined by the operation:

4 $${{\mathbb{T}}}_{\rm{S}}=\,\frac{1}{\sqrt{2}}\left[\begin{array}{cccc}1&0&1&0\\ 0&1&0&1\\ 1&0&-1&0\\ 0&1&0&-1\end{array}\right]$$

Following this transformation, we perform fourfold coincidence measurements between the output ports and the heralding photons, projecting the state shared between nodes A and H onto the maximally entangled target state $\left\vert \Psi \right\rangle =\frac{1}{\sqrt{2}}\left(\left\vert 01\right\rangle +{e}^{i\varphi }\left\vert 10\right\rangle \right)$, where $\varphi$ is a relative phase between the two sources introduced by the set-up.

This single-channel entanglement-swapping protocol can be extended to operate across two simultaneous channels for connecting two pairs of remote users. In this scenario, the state of each source is expressed as $\left\vert \phi \right\rangle =\frac{1}{\sqrt{2}}\left({\left\vert \phi \right\rangle }^{({\rm{ch}}1)}+{\left\vert \phi \right\rangle }^{({\rm{ch}}2)}\right)$, where ${\left\vert \phi \right\rangle }^{({\rm{ch}}1)}=\frac{1}{\sqrt{2}}\left(\left\vert 00\right\rangle +\left\vert 11\right\rangle \right)$ and ${\left\vert \phi \right\rangle }^{({\rm{ch}}2)}=\frac{1}{\sqrt{2}}\left(\left\vert 22\right\rangle +\left\vert 33\right\rangle \right)$. Consequently, the initial four-photon state can be written in the form of a four-dimensional entangled state given by:

5 $$\left\vert \Phi \right\rangle =\frac{1}{2}\left({\left\vert \phi \right\rangle }_{1}^{({\rm{ch}}1)}+{\left\vert \phi \right\rangle }_{1}^{({\rm{ch}}2)}\right)\otimes \left({\left\vert \phi \right\rangle }_{2}^{({\rm{ch}}1)}+{\left\vert \phi \right\rangle }_{2}^{({\rm{ch}}2)}\right),$$

where the subscript indicates the source. The unitary operation that allows us to swap entanglement simultaneously across the two channels is given by equation (1) in the main text.

After the transformation of the signal photon states, we perform two sets of fourfold coincidence measurements across the two network channels, projecting the states shared between distant nodes A and H onto two different maximally entangled target states: ${\left\vert \Psi \right\rangle }^{({\rm{ch}}1)}=\frac{1}{\sqrt{2}}\left(\left\vert 01\right\rangle -{e}^{i\varphi }\left\vert 10\right\rangle \right)$ for the state shared between A_1_H_1_, and ${\left\vert \Psi \right\rangle }^{({\rm{ch}}2)}=\frac{1}{\sqrt{2}}\left(\left\vert 23\right\rangle -{e}^{i\varphi }\left\vert 32\right\rangle \right)$ for the state shared between A_2_H_2_. As before, $\varphi$ represents the relative phase introduced by the set-up. This process allows us to simultaneously obtain two distinct swapped states between A_1_H_1_ and A_2_H_2_.

In both cases, the set of four-photon correlation measurements allows us to characterize swapped entangled states through quantum state tomography (refer to the next section). We determine the relative phase and reconstruct the estimated states for each scenario and channel, achieving fidelities to the target state $\left\vert \Psi \right\rangle$ of more than 77%, thus successfully validating our protocol.

### Quantum state tomography

Each realization of the SWAP gate in the programmable quantum network generates a bipartite quantum state between nodes A and H, upon which we perform full quantum state tomography. This entails measuring a tomographically complete set of measurements consisting of all local Pauli eigenvectors and performing Maximum-likelihood state estimation^36^. The complete set of measurements is given by ${\{{\Pi }_{ambn}^{\rm{AH}} = {\Pi }_{a| m}^{\rm{A}}\otimes {\Pi }_{b| n}^{\rm{H}}\}}_{abmn}$, where ${\Pi }_{a| m}^{\rm{A}}=\left\vert {v}_{a| m}\right\rangle \left\langle {v}_{a| m}\right\vert$ and *a* ∈ {1, −1} indexes the eigenvector of the Pauli matrix *σ*_*m*_ for *m* ∈ {*X*, *Y*, *Z*}. For each measurement, *n*_*ambn*_, four-photon coincidences are recorded and the maximum-likelihood estimator is obtained by iterating

6 $${\rho }_{k+1}={\mathscr{N}}[{\mathcal{R}}({\rho }_{k}){\rho }_{k}{\mathcal{R}}({\rho }_{k})],$$

where

7 $${\mathcal{R}}(\rho ):= \sum _{i \sim ambn}\frac{1}{{M}_{mn}}\frac{{n}_{i}}{{\rm{Tr}}[{\Pi }_{i}^{\rm{AH}}\rho ]}{\Pi }_{i}^{\rm{AH}},$$

with ${\rho }_{0}={\mathbb{I}}/d$ taken as the maximally mixed state, *M*_*mn*_ = ∑_*ab*_*n*_*ambn*_ are the four-photon events in each basis, and ${\mathscr{N}}[\rho ]:= \rho {\rm{Tr}}[\rho ]$ imposes unit trace. To estimate the statistical noise present in our state estimates, we perform Monte Carlo bootstrapping by taking Poissonian samples from our data and repeating the estimation procedure. We obtain 2,000 state estimates and their corresponding state fidelities to arrive at the one standard error stated in our presented state fidelities.

We perform quantum state tomography to recover the density matrix of the states shared by users in nodes A and H to demonstrate the success of our entanglement-swapping protocol. We estimate a fidelity to the two-dimensional maximally entangled target state of 88.1 ± 2.0% for single channel swapping. In the multiplexed case, we estimate fidelities of 77.1 ± 3.3% for the first channel (A_1_H_1_) and 83.2 ± 2.7% for the second channel (A_2_H_2_).

## Online content

Any methods, additional references, Nature Portfolio reporting summaries, source data, extended data, supplementary information, acknowledgements, peer review information; details of author contributions and competing interests; and statements of data and code availability are available at 10.1038/s41566-025-01806-x.

## Supplementary information

Supplementary Information Supplementary Discussion, which comprises Sections 1–5, Figs. 1–5 and Tables 1–3.

## Source data

Source Data Fig. 3 Zip file containing .txt files of data for Fig. 3 on multiplexed entanglement routing.

Source Data Fig. 4 Zip file containing .txt files of data for Fig. 4 on multiplexed entanglement swapping.

## Data Availability

Source data are provided with this paper.
