# Supplementary material for: A large-scale reconfigurable multiplexed quantum photonic network
Source: Nat Photonics. 2025 Nov 26;20(2):202–7. doi: 10.1038/s41566-025-01806-x (PMC12875861; doi:10.1038/s41566-025-01806-x)
Supplement: Supplementary file 1 — Supplementary Discussion, which comprises Sections 1–5, Figs. 1–5 and Tables 1–3. [file 41566_2025_1806_MOESM1_ESM.pdf]

nature photonics

Supplementary information https://doi.org/10.1038/s41566-025-01806-x

A large-scale reconfigurable multiplexed quantum photonic network

In the format provided by the authors and unedited

Supplementary information for: A Large-Scale Reconfigurable Programmable Quantum Photonic Network

The following supplementary information is provided: Description of the experimental setup (S.1), details of the preparation and characterisation of the entangled states entering the circuit (S.3), matrix representation of the 8×8-dimensional operations performed by the circuit (S.2), results for two and three-dimensional entanglement routing over a single channel (S.4), and performance of the circuit over time (S.5).

S.1. Detailed experimental setup

The experimental setup for our programmable network is illustrated in Fig S.1.

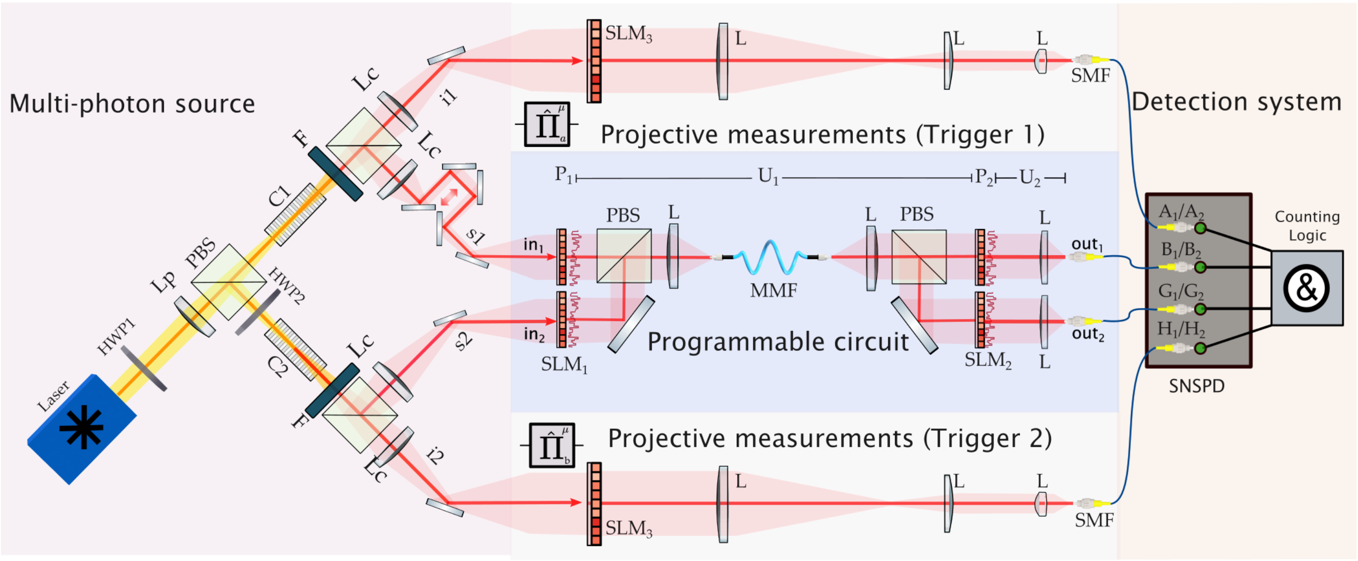

Figure S.1. **Experimental implementation** Two multi-user local networks are implemented by generating high-dimensional spatial-mode entanglement through spontaneous parametric down-conversion (SPDC) in periodically poled Potassium Titanyl Phosphate (ppKTP) crystals (C1 and C2). The signal photons from each pair (s_1 and s_2) are sent to users {B_1,B_2,G_1,G_2} through the programmable circuit composed of a multi-mode optical fibre (MMF) and two spatial light modulators divided into two screens each (SLM_1:$P_{1,2}$ and SLM_2:$P_{3,4}$). To ensure both input photons are indistinguishable through the circuit, identical paths are ensured with a delay stage on the path of s_1. Users {B_1,B_2,G_1,G_2} use phase screens P_3 and P_4 and single-mode fibres (SMFs) to perform projective measurements of their respective single-photon spatial modes. The other two photons from each entangled state (i_1 and i_2) are sent directly to users {A_1,A_2,H_1,H_2}, where projective measurements in spatial modes are performed by combining a spatial light modulator (SLM_3) and single-mode fibres (SMF). Note that SLM_3 is divided into two sections, each for manipulating a different idler photon. Photons arriving at each user pair are coupled to SMFs that guide them to superconducting nanowire single-photon detectors (SNSPD). Coincidence events between the eight users are registered through a time-tagging counting logic. L: lens, PBS: polarised beam-splitter, F: filter

S.2. Entangled photon sources for local networks

A 775-nm Ti:Sapphire femtosecond pulsed laser with a pulse duration of 140fs is divided into two beams with a polarised beam-splitter (PBS) and a half-wave plate (HWP1). Each beam is focused with a power of 750 mW onto a ppKTP crystal (1mm × 2mm × 5mm) to generate independent pairs of high-dimensional, transverse-spatial-mode entangled photons. After the crystal, the pump is dumped using a dichroic mirror, and the signal and idler photons from each source are separated using a PBS.

The beam waist ($1/e^2$ radius) of the pump at the centre of each crystal is set to w_p = 62μm with a lens L_p (f_p =150 mm). With this waist, we estimate the Schmidt number of each of the generated biphoton states to be $K_G \sim 6$ [1, 2]. Since the collection optics will further reduce the available entanglement, this modal bandwidth is an upper bound of the dimensionality we expect from each entangled pair.

To characterise the quality and entanglement dimensionality of the local states before manipulation by the circuit, we perform correlation measurements between photons generated at each source in the four-dimensional spatial-mode input bases defined for the multiplexed network implementation. These measurements are performed at the input of the multiport circuit, where we consider users B_1 and B_2 (G_1 and G_2) to be at input 1 (2), such that photon pairs from source S_1 are multiplexed and guided to users A_1, A_2 and B_1, B_2, while photon pairs from source S_2 are multiplexed and guided to users G_1, G_2 and H_1, H_2.

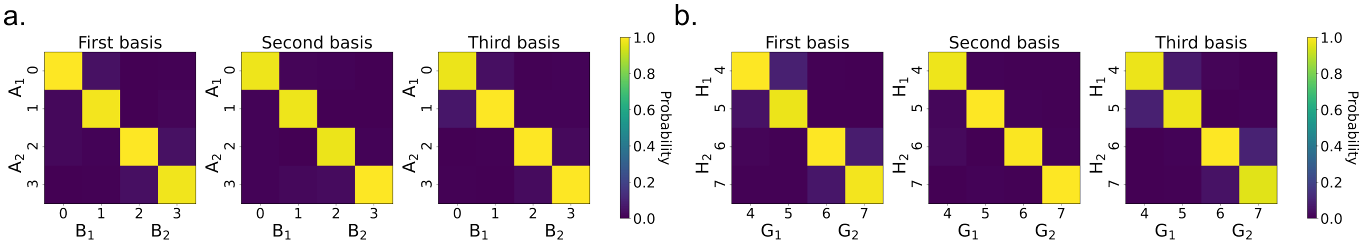

Figure S.2. **Input entangled states for multiplexed distribution**. Normalised two-photon correlations measured in three mutually unbiased bases (MUBs) for the biphoton states generated at sources a) S_1 (correlations between A and B) and b) S_2 (correlations between G and H).

Using the four-dimensional spatial-mode basis composed of macro-pixel modes $\{|m\rangle\}_m$, with $m = \{0,1,2,3\}$ labelling the modes for source 1 (S_1), and $m = \{4,5,6,7\}$ labelling the modes for source 2 (S_2), we split each mode set into two qubit subspaces (Ch1 and Ch2) and measure two-photon correlations between pairs originating at each source (See Fig. S.2). Using the correlations in all mutually unbiased bases between pairs A_1B_1 (Source 1, Ch 1), A_2B_2 (Source 1, Ch 2), G_1H_1 (Source 2, Ch 1), and G_2H_2 (Source 2, Ch 2), we can calculate the fidelity of the states input into each channel to the two-dimensional maximally entangled state via a fidelity witness [3]. As shown in the last two columns of Table S.1, all the input states have an average fidelity of 93.9%, demonstrating the high-quality qubit entanglement entering the network.

Table S.1: Fidelities to the maximally entangled state of the initial states generated at each source

Source	2-dimensional state) (single channel)	3-dimensional state (single channel)	2-dimensional state (multiplexed, Ch1)	2-dimensional state (multiplexed, Ch2)
S_1	$96.4 \pm 0.2\%$	$93.7 \pm 0.2\%$	$95.0 \pm 0.1\%$	$95.5 \pm 0.1\%$
S_2	$92.7 \pm 0.2\%$	$91.3 \pm 0.1\%$	$92.4 \pm 0.1\%$	$92.7 \pm 0.1\%$

*Errors are reported to one standard deviation

The energy and phase-matching conditions for our pump and crystal parameters lead to some non-separability in the joint-spectral amplitude (JSA), thus reducing heralded single-photon purity. This limits the Hong-Ou-Mandel interference visibility and the subsequent entanglement swapping fidelity. This reasoning is more nuanced when considering multiple spatial modes, whose JSAs may be distinct. However, our configuration minimises the spatial-spectral coupling across the spatial modes of interest [1] so the JSAs should be well approximated by the collinear JSA. A natural route to improving this purity would be to reduce the spectral bandwidth of the pump, decrease the crystal length, or introduce spectral bandpass filters on the photon(s) [4]. Domain-engineered crystals could also be employed to tailor the crystal nonlinearity, eliminating the need for narrowband spectral filtering. This would reduce losses while improving the overall signal-to-noise ratio [5]

An alternative route to spectral filtering originates from the finite spectral bandwidth of the scattering medium [6, 7]. Since the operations programmed in the MMF are optimised for the central wavelength at which it was classically characterised, the reduced mode transformation efficiency at more distant frequencies leads to effective spectral filtering into the desired output mode. This effect was sufficient to observe an approximately 10 nm (FWHM) Gaussian spectral filtering on our programmed identity gate, allowing us to obtain high-quality swapped state fidelities even after the removal of spectral bandpass filters used for the classical characterisation. The precise nature of these effects depends intimately on the spatial modes in use, the type of gate transformation implemented, the dispersive properties of the randomly configured MMF, as well as the spectral-spatial properties of the bi-photon state generated in the crystal. As such, the different effects that lead to a reduction of our swapped state fidelities cannot be exactly isolated.

For the network implementation, the signal photons s_1 and s_2 are injected into the optical circuit with orthogonal polarisations. To control their indistinguishability, we introduced a temporal delay with a motorised stage in the path of s_1. The idler photons i_1 and i_2 are manipulated with an SLM and SMF combination, allowing for single-outcome projective measurements of arbitrary spatial modes. Taking into account the physical parameters of the SPDC generation, we estimate the correlation bandwidth σ_S of the biphoton states at the Fourier plane and use a set of three lenses between crystal and SLMs to adequately collect and resize the pair of photons such that they cover most of the area of each SLM screen (600×400 pixels with 20 μm pixel pitch), but avoids any clipping. For the sake of simplicity, this optical system is represented in Fig S.1 by the lens L_c. After reflection from the SLMs, a telescope system and an aspheric lens are used for mode-matching the projected photons to either SMF or MMF collection modes (In Fig. S.1, this optical system is labelled as L inside the programmable circuit section, and shown explicitly with the three lenses in the projective measurements section).

S.3. 8×8-dimensional circuits implemented by the reconfigurable multiport device

We implement four different kinds of circuits defined by operations $\{\mathbb{T}_I, \mathbb{T}_X, \mathbb{T}_M, \mathbb{T}_S\}$, which are given by the matrices:

$$\mathbb{T}_I = \begin{bmatrix} 1&0&0&0&0&0&0&0\\ 0&1&0&0&0&0&0&0\\ 0&0&1&0&0&0&0&0\\ 0&0&0&1&0&0&0&0\\ 0&0&0&0&1&0&0&0\\ 0&0&0&0&0&1&0&0\\ 0&0&0&0&0&0&1&0\\ 0&0&0&0&0&0&0&1 \end{bmatrix}, \quad \mathbb{T}_X = \frac{1}{\sqrt{2}} \begin{bmatrix} 0&0&0&0&1&0&0&0\\ 0&0&0&0&0&1&0&0\\ 0&0&0&0&0&0&1&0\\ 0&0&0&0&0&0&0&1\\ 1&0&0&0&0&0&0&0\\ 0&1&0&0&0&0&0&0\\ 0&0&1&0&0&0&0&0\\ 0&0&0&1&0&0&0&0 \end{bmatrix}, \quad \mathbb{T}_M = \frac{1}{\sqrt{2}} \begin{bmatrix} 1&0&0&0&0&0&0&0\\ 0&1&0&0&0&0&0&0\\ 0&0&0&0&1&0&0&0\\ 0&0&0&0&0&1&0&0\\ 0&0&1&0&0&0&0&0\\ 0&0&0&1&0&0&0&0\\ 0&0&0&0&0&0&1&0\\ 0&0&0&0&0&0&0&1 \end{bmatrix}. \tag{S.3.1}$$

The operation for the multiplexed entanglement swapping protocol $\mathbb{T}_S$, as well as labels indicating the ordering of modes and channels are shown in Table S.2 below.

			In1				In2			
	Channels		Ch 1		Ch 2		Ch 1		Ch 2	
		Modes	0	1	2	3	4	5	6	7
Out 1	Ch 1	0	1	0	0	0	1	0	0	0
		1	0	1	0	0	0	1	0	0
	Ch 2	2	0	0	1	0	0	0	1	0
		3	0	0	0	1	0	0	0	1
Out 2	Ch 1	4	1	0	0	0	-1	0	0	0
		5	0	1	0	0	0	-1	0	0
	Ch 2	6	0	0	1	0	0	0	-1	0
		7	0	0	0	1	0	0	0	-1

Table S.2. Transformation between input and output modes over two channels implemented with operation $\mathbb{T}_S$.

S.4. Programmable routing of entanglement over a single channel

To route d-dimensional entanglement (with $d = 2, 3$) using a single channel, we will consider four users: Ada (A) and Barb (B), Grace (G) and Hedy (H), and input or local entangled states of the form $|\Phi\rangle = \frac{1}{\sqrt{d}} \sum_{i=0}^{d-1} |ii\rangle$. The set $\{|d\rangle\}_d$ forms a d-dimensional macro-pixel basis in which we will perform two-photon correlation measurements. For a chosen dimension d, we select the macro-pixel modes $\{|0\rangle_1, ..., |d\rangle_1, |0\rangle_2, ..., |d\rangle_2\}$, where the subscripts 1 and 2 indicate which input signal photon the modes correspond to.

First, we characterise the input entangled states $|\Phi\rangle$ with correlation measurements between the pair of photons generated at each source. These measurements are performed at the input of the multiport, where we consider users Barb (B) and Grace (G) to be at input 1 and 2 respectively, such that pairs of photons from source S_1 are guided

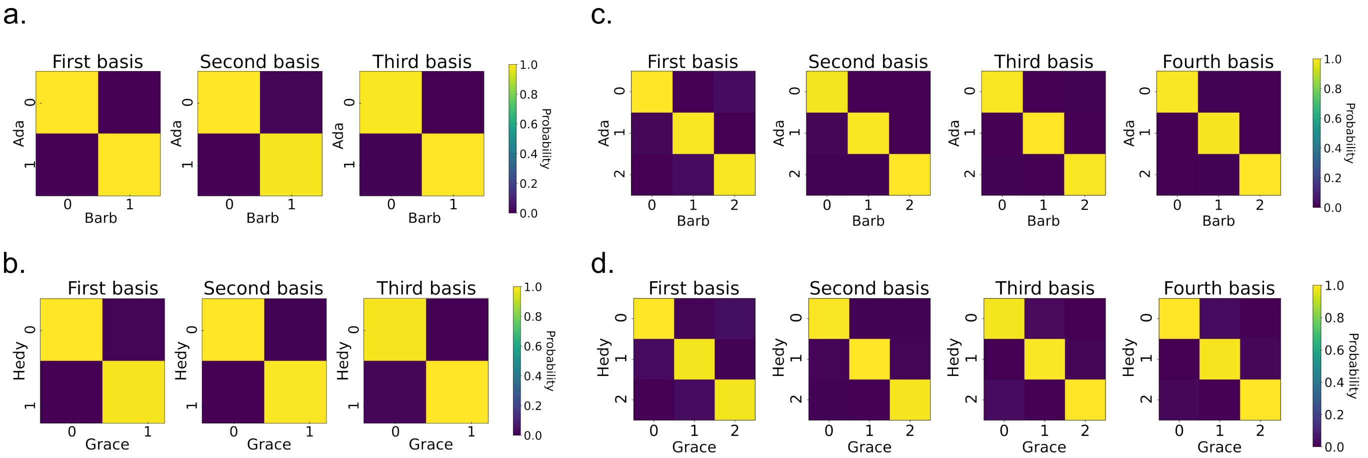

Figure S.3. **Two-dimensional and three-dimensional input entangled states for distribution over a single channel**. Two-photon correlation measurements in all mutually unbiased bases (MUBs) in dimensions $d = 2$ (a-b) and $d = 3$ (c-d) are made between pairs of photons generated at each source. Correlations between Ada and Barb (Grace and Hedy), shown on the top (bottom), correspond to states originating from source 1 (source 2).

directly to users Ada (A) and Barb (B), while pairs of photons from source S_2 are guided to users Grace (G) and Hedy (H). As described in Methods, we use two-fold coincidence measurements in all mutually unbiased bases (Fig. S.3) to estimate fidelities to the maximally entangled state in dimensions $d = 2, 3$ [3]. As shown in Table S.1, we can certify two-dimensional and three-dimensional entanglement at the input of the circuit with average fidelities of 94.6% and 92.5% respectively.

Once the input entangled states are characterised, we move to the single-channel network implementation. At the input side of the circuit, we use the d-dimensional macro-pixel basis $\{|0\rangle_1, ..., |d\rangle_1, |0\rangle_2, ..., |d\rangle_2\}$. For the output modes, we select d foci on each of the output ports of the circuit, which are directed to Barb and Grace. As before, single-outcome projective measurements on the d-dimensional macro-pixel basis over the idler photons herald the corresponding input modes on users Ada and Hedy.

The simultaneous distribution of qubit entanglement between AB and GH (AG and BH) is achieved with the 4x4 dimensional $\mathbb{I}$ ($\mathbb{X}$) gate. We can also program 6x6 dimensional $\mathbb{I}$ ($\mathbb{X}$) operations to share three-dimensional entanglement between AB and GH, or AG and BH.

To certify the distributed entangled states, we perform two-fold coincidence measurements in all mutually unbiased bases between pairs of nodes (Fig. S.4) and estimate fidelities to the maximally entangled state in dimensions $d = 2, 3$ [3]. As shown in Table S.3, the obtained fidelities violate the dimensionality bounds, allowing us to certify the routing of two and three-dimensional entanglement over the network.

Table S.3: Fidelities to the maximally entangled state of the states shared in the programmable network using a single channel

Gate	2-dim states		3-dim states	
$\mathbb{I}$	AB	GH	AB	GH
	86.2 ± 1.1%	83.1 ± 1.1%	73.1 ± 1.8%	77.0 ± 1.5%
$\mathbb{X}$	AG	BH	AG	BH
	81.2 ± 1.2%	83.6 ± 1.2%	77.4 ± 1.8%	74.7 ± 1.8%
SWAP	AH		-	
	88.1 ± 2.0		-	

*Errors are reported to three standard deviations

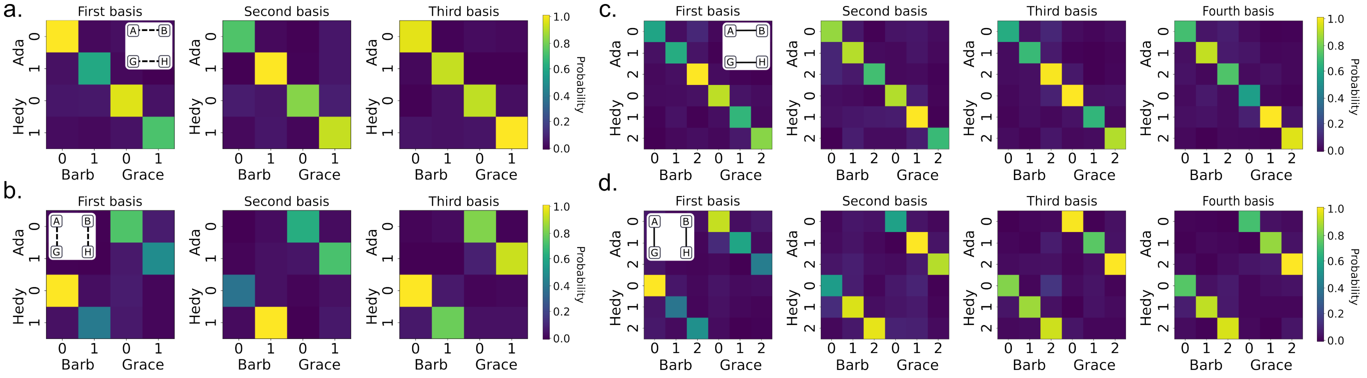

Figure S.4. **Two-dimensional and three-dimensional entanglement routing**. We distribute entanglement between two users via a single channel either with a two-dimensional entangled state (a,b) or a three-dimensional state (c,d). We perform measurements in all mutually unbiased bases to characterise the operation of each configuration. The correlation matrices correspond to two-photon coincidence measurements, showcasing the circuit's operation.

S.5. Circuit stability

We study the stability of the complex media-based circuit by characterising the performance of the programmable circuit over time. With a single initial characterisation of the MMF transmission matrix and without performing any experimental realignment, we regularly test our highest-dimensional gates (8×8) over 14 days. Fidelity estimates of the states shared over the two channels are obtained through two-fold coincidence measurements performed in all mutually unbiased bases. For each programmed gate, we record a dataset and then run 5000 iterations of a Monte Carlo simulation assuming Poissonian photon-counting statistics. From this, we extract the mean fidelity over all channels of the entangled states shared with Ada (or Hedy), together with an error of one standard deviation. The results are shown in Fig. S.5 and consistently exceed the bound for a two-dimensional separable state. The consistent certification of the multiplexed and adaptable entanglement routing over time demonstrates the robustness of our platform to temperature (±0.5 °C) and mechanical fluctuations in the lab.

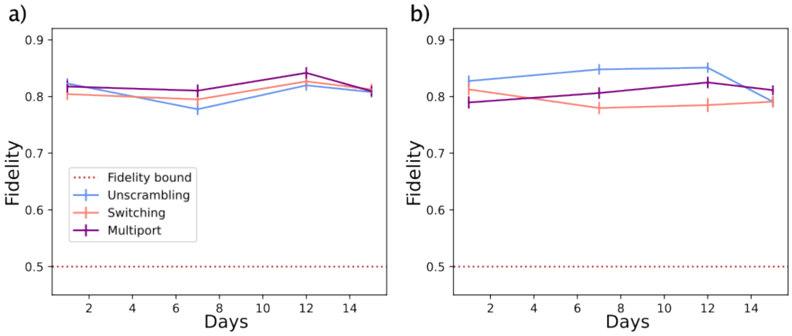

Figure S.5. **Entanglement routing stability**. We measure the fidelities of the states shared through the three different 8x8 unitary gates to the two-dimensional maximally entangled state, implemented continuously over a period of two weeks. Average fidelities over the two channels are shown for a) states shared with node Ada, and b) states shared with node Hedy. Entanglement is either routed between local users AB and GH (Unscrambling) with gate $\mathbb{T}_I$, between global users AG and BH (Switching) with gate $\mathbb{T}_S$, or shared across both local and global networks with $\mathbb{T}_M$ (Multiport). The red dotted line indicates the fidelity bound for a separable state. As indicated in the inset legend, each point represents the mean fidelity over the two channels, with vertical error bars showing an error of one standard deviation.

[1] Vatshal Srivastav, Natalia Herrera Valencia, Saroch Leedumrongwatthanakun, Will McCutcheon, and Mehul Malik, *Characterizing and tailoring spatial correlations in multimode parametric down-conversion,* Physical Review Applied **18**, 054006 (2022), arxiv:2110.03462.

[2] C. K. Law and J. H. Eberly, *Analysis and interpretation of high transverse entanglement in optical parametric down conversion,* Physical Review Letters **92**, 127903 (2004).

[3] Jessica Bavaresco, Natalia Herrera Valencia, Claude Klöckl, Matej Pivoluska, Paul Erker, Nicolai Friis, Mehul Malik, and Marcus Huber, *Measurements in two bases are sufficient for certifying high-dimensional entanglement,* Nature Physics **14**, 1032 (2017), arXiv:1709.07344.

[4] Francesco Graffitti, Jérémy Kelly-Massicotte, Alessandro Fedrizzi, and Agata M. Brańczyk, *Design considerations for high-purity heralded single-photon sources,* Physical Review A **98**, 053811 (2018).

[5] Francesco Graffitti, Peter Barrow, Massimiliano Proietti, Dmytro Kundys, and Alessandro Fedrizzi, *Independent high-purity photons created in domain-engineered crystals,* Optica **5**, 514 (2018), arXiv:1712.07140.

[6] Daria Andreoli, Giorgio Volpe, Sébastien Popoff, Ori Katz, Samuel Grésillon, and Sylvain Gigan, *Deterministic control of broadband light through a multiply scattering medium via the multispectral transmission matrix,* Scientific Reports **5** (2015), arXiv:1412.0368.

[7] Joel Carpenter, Benjamin J. Eggleton, and Jochen Schröder, *Complete spatiotemporal characterization and optical transfer matrix inversion of a 420 mode fiber,* Optics Letters **41**, 5580 (2016).
